# Crystal structure of 3-amino­pyridinium 1′-carb­oxy­ferrocene-1-carboxyl­ate

**DOI:** 10.1107/S2056989017007058

**Published:** 2017-05-16

**Authors:** Aleksei V. Medved’ko, Andrei V. Churakov, Haojie Yu, Wang Li, Sergey Z. Vatsadze

**Affiliations:** a Department of Chemistry, M.V. Lomonosov Moscow State University, Leninskie Gory 1/3, Moscow 119991, Russian Federation; b Institute of General and Inorganic Chemistry, Russian Academy of Sciences, Leninskii prosp. 31, Moscow 119991, Russian Federation; c State Key Laboratory of Chemical Engineering, College of Chemical and Biological Engineering, Zhejiang University, Hangzhou 310027, People’s Republic of China

**Keywords:** crystal structure, ferrocene-1,1′-di­carb­oxy­lic acid, ferrocene conformation

## Abstract

The structure of the title salt, (C_5_H_7_N_2_)[Fe(C_6_H_4_O_2_)(C_6_H_5_O_2_)], consists of 3-amino­pyridinium cations and 1′-carb­oxy­ferrocene-1-carboxyl­ate monoanions. The ferrocenyl moiety of the anion adopts a typical sandwich structure, with Fe—C distances in the range 2.0270 (15)–2.0568 (17) Å. The anion possesses an eclipsed conformation, with the torsion angle φ (C_subst_—Cp_cent_—Cp_cent_— C_subst_) equal to 66.0°. The conformations of other 1′-carb­oxy­ferrocene-1-carboxyl­ate monoanions are compared and analyzed on the basis of literature data.

## Abstract 2

The title structure consists of 3-amino­pyridinium cations and 1′-carb­oxy­ferrocene-1-carboxyl­ate monoanions held together by N—H⋯O and O—H⋯O hydrogen bonds.

## Chemical context   

The idea behind this research was to use ferrocenedi­carb­oxy­lic acid as a dianionic building block in supra­molecular polymer and conventional polymer design (Amer *et al.*, 2013 ▸; Sun *et al.*, 2016 ▸; Zheng *et al.*, 2016 ▸).
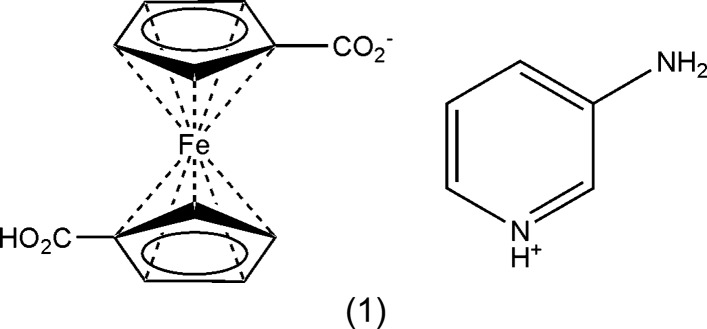


We tried to apply the trio of available amino­pyridines, namely 2-, 3- and 4-amino­pyridine, as basic counterparts to ferrocenedi­carb­oxy­lic diacid. One of the ideas was to check the possibility of obtaining gels with a supra­molecular arrangement of the constituents in alcoholic media. All those reactions were carried out in a 1:2 ratio of acid–amine in order to exploit both carb­oxy­lic acid groups of the diacid. The experiments revealed, however, that while in cases of 2- and 4-amino­pyridine, only amorphous powders could be obtained, the reaction of 3-amino­pyridine led to a crystalline salt, 3-amino­pyridinium 1′-carb­oxy­ferrocene-1-carboxyl­ate, (1), but with a 1:1 composition.

## Structural commentary   

**Figure 1:**
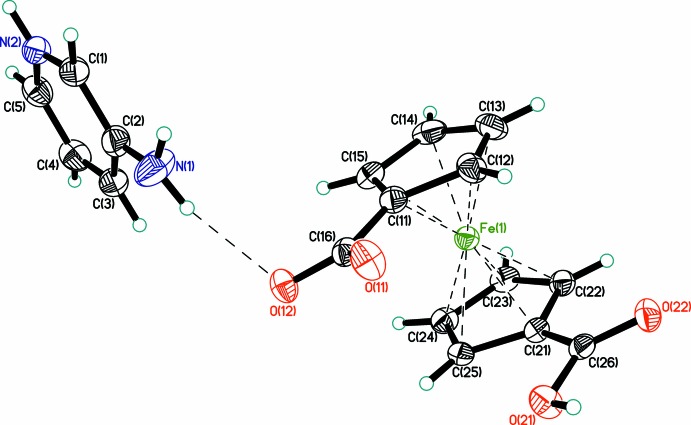
The structures of the mol­ecular components in (1). Displacement ellipsoids are shown at the 50% probability level. Hydrogen bonding is shown as dashed lines.

**Figure 2:**
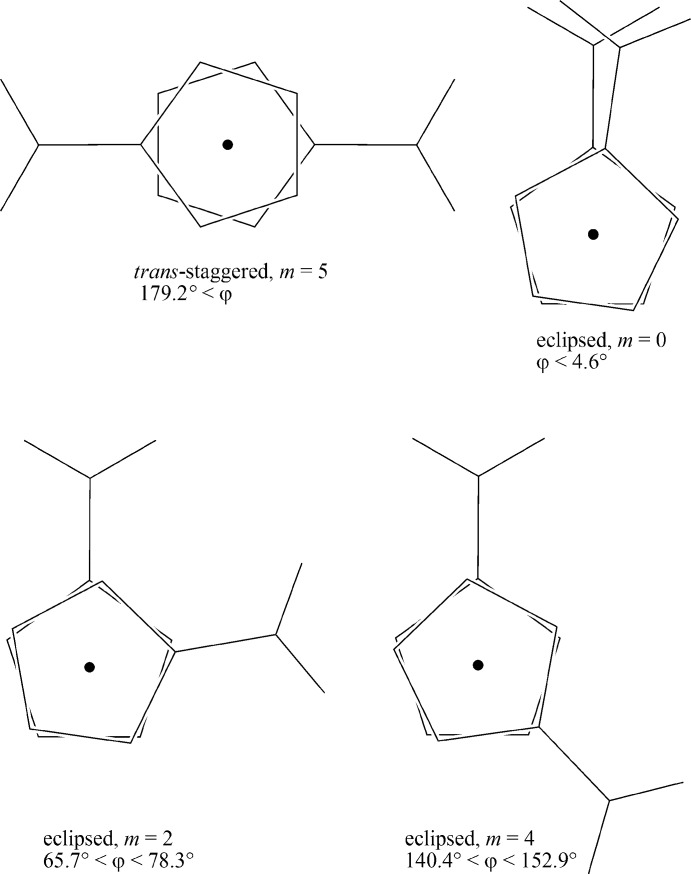
The four known conformations of the (HO_2_C-η^5^-C_5_H_4_)Fe(η^5^-C_5_H_4_—CO_2_^−^) anion.

The crystal structure of (1) consists of one 3-amino­pyridinium cation and one 1′-carb­oxy­ferrocene-1-carboxyl­ate monoanion (Fig. 1 ▸). In the cation, the pyridine N atom is protonated. The ferrocenyl moieties adopt the characteristic sandwich structure, with typical Fe—C distances in the range 2.0270 (15)–2.0568 (17) Å (Table 1 ▸). The Fe^II^ atom is slightly (∼0.01 Å) shifted towards the substituted C11 and C21 atoms. The C16—O bond lengths within the carboxyl­ate anion are almost equal [1.2604 (19) and 1.2636 (19) Å], whereas, in contrast, they differ greatly within the carb­oxy­lic acid group, with C26=O22 = 1.2128 (19) Å and C26—O21 = 1.326 (2) Å, the latter involving the OH group. The planes of the cyclo­penta­dienyl (Cp) rings are almost parallel to the planes of the corresponding carb­oxy/carboxyl­ate groups, with O—C—C—C torsion angles less than 13°. The conformation of 1,1′-disubstituted ferrocenes is described by the torsion angle C_subst_—Cp_cent_—Cp_cent_—C_subst_, where C_subst_ stands for a ferrocene C atom with an additional bonding partner and Cp_cent_ for the centre of gravity of the C atoms of the ring; this angle is hereafter referred to as φ. In (1), the anion possesses an eclipsed conformation with φ = 66.0° (ideal value 72°) (Fig. 2 ▸).

## Supra­molecular features   

**Figure 3:**
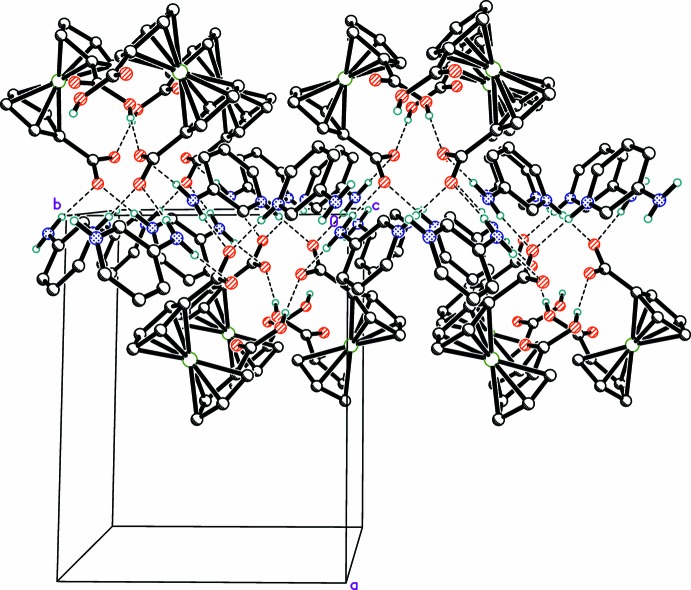
The formation of hydrogen-bonded layers parallel to (100) in the crystal. Hydrogen bonds are drawn as dashed lines.

In the title crystal, adjacent cationic and anionic units are combined into a layered arrangement parallel to (100) by charge-supported NH⋯^−^O_2_C hydrogen bonds of medium–strong-to-weak nature and of CO_2_H⋯^−^O_2_C hydrogen bonds of strong nature (Table 2 ▸ and Fig. 3 ▸).

## Database survey   

**Figure 4:**
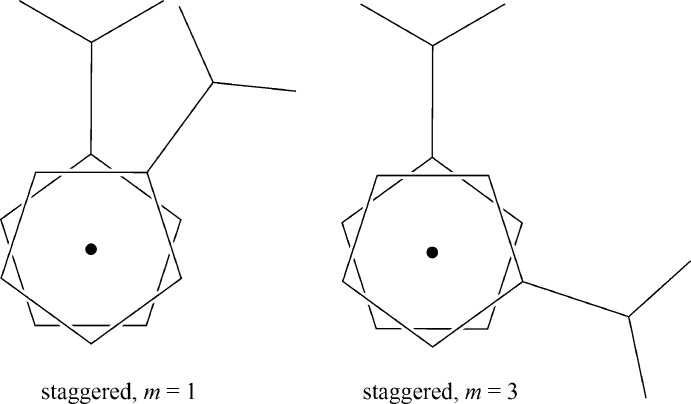
Unobserved staggered conformations in the structures containing a 1′-carb­oxy­ferrocene-1-carboxyl­ate acid monoanion.

The Cambridge Structural Database (CSD, Version 5.38 of February 2017; Groom *et al.*, 2016 ▸) contains data for 11 structures comprising (HO_2_C-η^5^-C_5_H_4_)Fe(η^5^-C_5_H_4_-CO_2_^−^) units from 14 crystallographically independent monoanions. Among these 14 fragments, three adopt a *trans*-staggered conformation, with *m* = 5 (as defined in Zakaria *et al.*, 2002 ▸). Others adopt three eclipsed conformations with *m* = 0, 2 and 4 (3, 4 and 4 cases, respectively; Fig. 2 ▸). Surprisingly, two staggered conformations with *m* = 1 and 3 (Fig. 4 ▸) were not observed.

## Synthesis and crystallization   

### Preparation of ferrocene-1,1′-di­carb­oxy­lic acid (Gao *et al.*, 2009 ▸)   

An 8% NaOCl aqueous solution (100 ml) was added dropwise to 1,1′-di­acetyl­ferrocene (5.37 g, 20 mmol) under stirring at a temperature of 317–320 K. The solution was stirred at this temperature for 2 h. Three more 25 ml portions of NaOCl solution were added every 2 h. The reaction mixture was filtered and acidified to a pH of 1.1 with 10% hydro­chloric acid and cooled to 277 K overnight. The yellow precipitate which formed was filtered off and recrystallized from ethanol to give an orange microcrystalline powder (yield 2.18 g, 40%).

### Preparation of 3-amino­pyridinium 1′-carb­oxy­ferrocene-1-carboxyl­ate, (1)   

Ferrocene-1,1′-di­carb­oxy­lic acid (50 mg, 0.18 mmol) was dissolved in methanol and mixed with a methano­lic solution of 3-amino­pyridine (33.8 mg, 0.36 mmol). The reaction mixture was filtered and subjected to slow evaporation at room temperature to give orange crystals of the title salt.

## Refinement   

Crystal data, data collection and structure refinement details are summarized in Table 3 ▸. All H atoms were located from a difference Fourier synthesis and refined isotropically without constraints or restraints.

## Supplementary Material

Crystal structure: contains datablock(s) I. DOI: 10.1107/S2056989017007058/wm5388sup1.cif

Structure factors: contains datablock(s) I. DOI: 10.1107/S2056989017007058/wm5388Isup2.hkl

CCDC reference: 1444115

Additional supporting information:  crystallographic information; 3D view; checkCIF report

## Figures and Tables

**Table 1:** Selected geometric parameters (Å, °)

Fe1—C21	2.0270 (15)	Fe1—C24	2.0515 (16)
Fe1—C15	2.0341 (16)	Fe1—C13	2.0517 (18)
Fe1—C11	2.0359 (16)	Fe1—C23	2.0568 (17)
Fe1—C22	2.0414 (17)	O11—C16	1.2604 (19)
Fe1—C25	2.0451 (16)	O12—C16	1.2636 (19)
Fe1—C12	2.0459 (17)	O21—C26	1.326 (2)
Fe1—C14	2.0496 (17)	O22—C26	1.2128 (19)
			
C12—C11—C16—O11	14.1 (2)	C25—C21—C26—O21	10.2 (2)

**Table 2:** Hydrogen-bond geometry (Å, °)

*D*—H⋯*A*	*D*—H	H⋯*A*	*D*⋯*A*	*D*—H⋯*A*
N1—H11⋯O12	0.87 (3)	2.08 (3)	2.918 (2)	161 (2)
N1—H10⋯O11^i^	0.84 (3)	2.07 (3)	2.906 (2)	171 (3)
N2—H2⋯O11^ii^	0.89 (2)	1.79 (2)	2.675 (2)	177 (2)
O21—H21⋯O12^iii^	0.81 (2)	1.77 (2)	2.5621 (16)	164 (2)

Symmetry codes: (i) 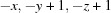; (ii) 
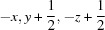
; (iii) 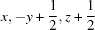.

**Table 3:** Experimental details

Crystal data	
Chemical formula	(C_5_H_7_N_2_)[Fe(C_6_H_4_O_2_)(C_6_H_5_O_2_)]
*M*_r_	368.17
Crystal system, space group	Monoclinic, *P*2_1_/*c*
Temperature (K)	150
*a*, *b*, *c* (Å)	13.2246 (10), 10.3040 (8), 11.7402 (9)
β (°)	101.703 (1)
*V* (Å^3^)	1566.5 (2)
*Z*	4
Radiation type	Mo *K*α
μ (mm^−1^)	0.99
Crystal size (mm)	0.22 × 0.20 × 0.02

Data collection	
Diffractometer	Bruker SMART APEXII
Absorption correction	Multi-scan (*SADABS*; Bruker, 2008 ▸)
*T*_min_, *T*_max_	0.812, 0.981
No. of measured, independent and observed [*I* > 2σ(*I*)] reflections	14616, 3409, 2812
*R*_int_	0.026
(sin θ/λ)_max_ (Å^−1^)	0.638

Refinement	
*R*[*F* ^2^ > 2σ(*F* ^2^)], *wR*(*F* ^2^), *S*	0.027, 0.073, 1.03
No. of reflections	3409
No. of parameters	281
H-atom treatment	All H-atom parameters refined
Δρ_max_, Δρ_min_ (e Å^−3^)	0.37, −0.23

Computer programs: *APEX2* and *SAINT* (Bruker, 2008 ▸) and *SHELXTL* (Sheldrick, 2008 ▸).

## supplementary crystallographic information

### Crystal data

**Table:**

(C_5_H_7_N_2_)[Fe(C_6_H_4_O_2_)(C_6_H_5_O_2_)]	*F*(000) = 760
*M**_r_* = 368.17	*D*_x_ = 1.561 Mg m^−^^3^
Monoclinic, *P*2_1_/*c*	Mo *K*α radiation, λ = 0.71073 Å
Hall symbol: -P 2ybc	Cell parameters from 5525 reflections
*a* = 13.2246 (10) Å	θ = 2.5–30.1°
*b* = 10.3040 (8) Å	µ = 0.99 mm^−^^1^
*c* = 11.7402 (9) Å	*T* = 150 K
β = 101.703 (1)°	Plate, orange
*V* = 1566.5 (2) Å^3^	0.22 × 0.20 × 0.02 mm
*Z* = 4	

### Data collection

**Table:**

Bruker SMART APEXII diffractometer	3409 independent reflections
Radiation source: fine-focus sealed tube	2812 reflections with *I* > 2σ(*I*)
Graphite monochromator	*R*_int_ = 0.026
ω scans	θ_max_ = 27.0°, θ_min_ = 2.5°
Absorption correction: multi-scan (*SADABS*; Bruker, 2008)	*h* = −16→16
*T*_min_ = 0.812, *T*_max_ = 0.981	*k* = −13→13
14616 measured reflections	*l* = −14→14

### Refinement

**Table:**

Refinement on *F*^2^	Primary atom site location: structure-invariant direct methods
Least-squares matrix: full	Secondary atom site location: difference Fourier map
*R*[*F*^2^ > 2σ(*F*^2^)] = 0.027	Hydrogen site location: difference Fourier map
*wR*(*F*^2^) = 0.073	All H-atom parameters refined
*S* = 1.03	*w* = 1/[σ^2^(*F*_o_^2^) + (0.0395*P*)^2^ + 0.5357*P*] where *P* = (*F*_o_^2^ + 2*F*_c_^2^)/3
3409 reflections	(Δ/σ)_max_ = 0.001
281 parameters	Δρ_max_ = 0.37 e Å^−^^3^
0 restraints	Δρ_min_ = −0.23 e Å^−^^3^

### Special details

**Table:**

Geometry. All esds (except the esd in the dihedral angle between two l.s. planes) are estimated using the full covariance matrix. The cell esds are taken into account individually in the estimation of esds in distances, angles and torsion angles; correlations between esds in cell parameters are only used when they are defined by crystal symmetry. An approximate (isotropic) treatment of cell esds is used for estimating esds involving l.s. planes.
Refinement. Refinement of F^2^ against ALL reflections. The weighted R-factor wR and goodness of fit S are based on F^2^, conventional R-factors R are based on F, with F set to zero for negative F^2^. The threshold expression of F^2^ > 2sigma(F^2^) is used only for calculating R-factors(gt) etc. and is not relevant to the choice of reflections for refinement. R-factors based on F^2^ are statistically about twice as large as those based on F, and R- factors based on ALL data will be even larger.

### Fractional atomic coordinates and isotropic or equivalent isotropic displacement parameters (Å^2^)

**Table:**

	*x*	*y*	*z*	*U*_iso_*/*U*_eq_	
Fe1	0.374096 (17)	0.50216 (2)	0.671077 (19)	0.01937 (8)	
O11	0.09010 (9)	0.36772 (12)	0.61033 (10)	0.0311 (3)	
O12	0.16389 (8)	0.34091 (11)	0.45865 (10)	0.0257 (3)	
O21	0.30237 (9)	0.21837 (12)	0.84337 (11)	0.0277 (3)	
O22	0.35822 (10)	0.38212 (12)	0.96535 (10)	0.0291 (3)	
C11	0.22302 (12)	0.51243 (15)	0.58942 (14)	0.0228 (3)	
C12	0.23757 (13)	0.57474 (17)	0.70015 (15)	0.0272 (4)	
C13	0.30979 (14)	0.67653 (17)	0.70209 (17)	0.0321 (4)	
C14	0.34025 (14)	0.67928 (17)	0.59322 (17)	0.0311 (4)	
C15	0.28745 (13)	0.57799 (16)	0.52300 (15)	0.0254 (4)	
C16	0.15563 (12)	0.39937 (16)	0.55094 (13)	0.0219 (3)	
C21	0.42157 (12)	0.36219 (15)	0.79136 (13)	0.0210 (3)	
C22	0.49364 (13)	0.46741 (16)	0.80757 (15)	0.0234 (3)	
C23	0.53193 (13)	0.48018 (16)	0.70361 (16)	0.0255 (4)	
C24	0.48348 (13)	0.38427 (16)	0.62345 (15)	0.0248 (3)	
C25	0.41579 (12)	0.31077 (15)	0.67722 (14)	0.0214 (3)	
C26	0.35821 (12)	0.32398 (15)	0.87553 (13)	0.0212 (3)	
H12	0.2043 (15)	0.5490 (19)	0.7600 (17)	0.029 (5)*	
H13	0.3336 (15)	0.727 (2)	0.7642 (18)	0.037 (5)*	
H14	0.3905 (16)	0.737 (2)	0.5730 (17)	0.037 (5)*	
H15	0.2915 (15)	0.555 (2)	0.4446 (18)	0.035 (5)*	
H21	0.2655 (18)	0.207 (2)	0.8897 (19)	0.048 (7)*	
H22	0.5128 (15)	0.5176 (18)	0.8710 (18)	0.030 (5)*	
H23	0.5770 (15)	0.543 (2)	0.6893 (16)	0.030 (5)*	
H24	0.4920 (14)	0.3759 (18)	0.5428 (18)	0.030 (5)*	
H25	0.3712 (14)	0.2455 (18)	0.6409 (15)	0.023 (5)*	
N1	0.04432 (16)	0.4901 (2)	0.26631 (16)	0.0457 (5)	
N2	0.03611 (12)	0.69407 (15)	0.01507 (13)	0.0281 (3)	
C1	0.01186 (13)	0.64261 (17)	0.11058 (15)	0.0288 (4)	
C2	0.07267 (14)	0.54610 (19)	0.17299 (15)	0.0303 (4)	
C3	0.16037 (14)	0.50701 (18)	0.13113 (17)	0.0324 (4)	
C4	0.18291 (14)	0.56395 (19)	0.03310 (17)	0.0340 (4)	
C5	0.11970 (14)	0.65834 (19)	−0.02507 (17)	0.0321 (4)	
H1	−0.0489 (15)	0.6761 (19)	0.1314 (16)	0.030 (5)*	
H2	−0.0059 (17)	0.753 (2)	−0.0242 (19)	0.044 (6)*	
H3	0.2027 (15)	0.446 (2)	0.1703 (17)	0.033 (5)*	
H4	0.2411 (17)	0.538 (2)	0.0051 (19)	0.041 (6)*	
H5	0.1301 (16)	0.700 (2)	−0.0915 (18)	0.039 (6)*	
H10	0.000 (2)	0.529 (3)	0.296 (2)	0.058 (8)*	
H11	0.0872 (19)	0.439 (2)	0.311 (2)	0.049 (6)*	

### Atomic displacement parameters (Å^2^)

**Table:**

	*U*^11^	*U*^22^	*U*^33^	*U*^12^	*U*^13^	*U*^23^
Fe1	0.01955 (13)	0.01805 (13)	0.02010 (13)	−0.00017 (9)	0.00306 (9)	0.00002 (9)
O11	0.0287 (6)	0.0375 (7)	0.0308 (6)	−0.0076 (5)	0.0146 (5)	−0.0072 (5)
O12	0.0222 (6)	0.0317 (6)	0.0240 (6)	−0.0020 (5)	0.0063 (5)	−0.0074 (5)
O21	0.0306 (6)	0.0273 (6)	0.0291 (6)	−0.0060 (5)	0.0153 (5)	−0.0031 (5)
O22	0.0395 (7)	0.0296 (6)	0.0189 (6)	−0.0017 (5)	0.0073 (5)	−0.0024 (5)
C11	0.0204 (7)	0.0244 (8)	0.0233 (8)	0.0044 (6)	0.0035 (6)	−0.0004 (6)
C12	0.0243 (8)	0.0291 (9)	0.0281 (9)	0.0045 (7)	0.0051 (7)	−0.0057 (7)
C13	0.0309 (9)	0.0233 (9)	0.0389 (10)	0.0050 (7)	−0.0008 (8)	−0.0088 (8)
C14	0.0290 (9)	0.0203 (8)	0.0417 (10)	0.0020 (7)	0.0015 (8)	0.0071 (8)
C15	0.0242 (8)	0.0253 (8)	0.0255 (9)	0.0020 (7)	0.0020 (7)	0.0059 (7)
C16	0.0182 (7)	0.0256 (8)	0.0211 (8)	0.0037 (6)	0.0026 (6)	0.0003 (6)
C21	0.0218 (7)	0.0202 (8)	0.0202 (8)	0.0020 (6)	0.0026 (6)	0.0015 (6)
C22	0.0214 (8)	0.0225 (8)	0.0242 (8)	−0.0007 (6)	−0.0004 (6)	0.0003 (7)
C23	0.0204 (8)	0.0245 (9)	0.0316 (9)	−0.0005 (6)	0.0053 (7)	0.0045 (7)
C24	0.0243 (8)	0.0259 (8)	0.0256 (9)	0.0040 (7)	0.0083 (7)	0.0019 (7)
C25	0.0233 (8)	0.0186 (8)	0.0226 (8)	0.0028 (6)	0.0050 (6)	−0.0008 (6)
C26	0.0221 (8)	0.0207 (7)	0.0199 (8)	0.0043 (6)	0.0021 (6)	0.0034 (6)
N1	0.0483 (11)	0.0575 (12)	0.0334 (9)	0.0270 (9)	0.0135 (8)	0.0118 (9)
N2	0.0255 (7)	0.0261 (8)	0.0313 (8)	0.0006 (6)	0.0024 (6)	−0.0017 (6)
C1	0.0254 (9)	0.0313 (9)	0.0292 (9)	0.0046 (7)	0.0047 (7)	−0.0042 (7)
C2	0.0296 (9)	0.0342 (9)	0.0260 (9)	0.0049 (8)	0.0028 (7)	−0.0042 (7)
C3	0.0280 (9)	0.0323 (10)	0.0343 (10)	0.0078 (8)	0.0001 (8)	−0.0053 (8)
C4	0.0243 (9)	0.0366 (10)	0.0422 (11)	0.0010 (8)	0.0096 (8)	−0.0093 (9)
C5	0.0295 (9)	0.0324 (10)	0.0356 (10)	−0.0045 (8)	0.0092 (8)	−0.0060 (8)

### Geometric parameters (Å, º)

**Table:**

Fe1—C21	2.0270 (15)	C21—C25	1.429 (2)
Fe1—C15	2.0341 (16)	C21—C22	1.431 (2)
Fe1—C11	2.0359 (16)	C21—C26	1.473 (2)
Fe1—C22	2.0414 (17)	C22—C23	1.419 (2)
Fe1—C25	2.0451 (16)	C22—H22	0.90 (2)
Fe1—C12	2.0459 (17)	C23—C24	1.424 (2)
Fe1—C14	2.0496 (17)	C23—H23	0.92 (2)
Fe1—C24	2.0515 (16)	C24—C25	1.415 (2)
Fe1—C13	2.0517 (18)	C24—H24	0.98 (2)
Fe1—C23	2.0568 (17)	C25—H25	0.938 (19)
O11—C16	1.2604 (19)	N1—C2	1.357 (3)
O12—C16	1.2636 (19)	N1—H10	0.84 (3)
O21—C26	1.326 (2)	N1—H11	0.87 (3)
O21—H21	0.81 (2)	N2—C1	1.337 (2)
O22—C26	1.2128 (19)	N2—C5	1.338 (2)
C11—C12	1.427 (2)	N2—H2	0.89 (2)
C11—C15	1.435 (2)	C1—C2	1.391 (3)
C11—C16	1.481 (2)	C1—H1	0.95 (2)
C12—C13	1.416 (3)	C2—C3	1.407 (3)
C12—H12	0.94 (2)	C3—C4	1.377 (3)
C13—C14	1.416 (3)	C3—H3	0.91 (2)
C13—H13	0.90 (2)	C4—C5	1.371 (3)
C14—C15	1.422 (3)	C4—H4	0.94 (2)
C14—H14	0.96 (2)	C5—H5	0.92 (2)
C15—H15	0.96 (2)		
			
C21—Fe1—C15	155.49 (7)	C13—C14—H14	125.0 (12)
C21—Fe1—C11	120.05 (6)	C15—C14—H14	126.7 (12)
C15—Fe1—C11	41.30 (7)	Fe1—C14—H14	124.5 (12)
C21—Fe1—C22	41.17 (6)	C14—C15—C11	107.83 (15)
C15—Fe1—C22	161.90 (7)	C14—C15—Fe1	70.20 (10)
C11—Fe1—C22	155.29 (7)	C11—C15—Fe1	69.42 (9)
C21—Fe1—C25	41.07 (6)	C14—C15—H15	128.3 (12)
C15—Fe1—C25	120.11 (7)	C11—C15—H15	123.9 (12)
C11—Fe1—C25	107.37 (6)	Fe1—C15—H15	126.2 (12)
C22—Fe1—C25	69.07 (7)	O11—C16—O12	122.66 (15)
C21—Fe1—C12	107.64 (7)	O11—C16—C11	118.27 (14)
C15—Fe1—C12	68.83 (7)	O12—C16—C11	119.03 (14)
C11—Fe1—C12	40.94 (6)	C25—C21—C22	108.26 (14)
C22—Fe1—C12	120.15 (7)	C25—C21—C26	126.92 (15)
C25—Fe1—C12	125.97 (7)	C22—C21—C26	124.58 (14)
C21—Fe1—C14	162.37 (7)	C25—C21—Fe1	70.14 (9)
C15—Fe1—C14	40.76 (7)	C22—C21—Fe1	69.95 (9)
C11—Fe1—C14	68.84 (7)	C26—C21—Fe1	121.30 (11)
C22—Fe1—C14	124.79 (7)	C23—C22—C21	107.47 (15)
C25—Fe1—C14	155.14 (7)	C23—C22—Fe1	70.32 (10)
C12—Fe1—C14	68.20 (8)	C21—C22—Fe1	68.87 (9)
C21—Fe1—C24	68.45 (6)	C23—C22—H22	124.7 (13)
C15—Fe1—C24	107.50 (7)	C21—C22—H22	127.9 (13)
C11—Fe1—C24	125.54 (7)	Fe1—C22—H22	126.3 (13)
C22—Fe1—C24	68.50 (7)	C22—C23—C24	108.23 (15)
C25—Fe1—C24	40.42 (6)	C22—C23—Fe1	69.16 (9)
C12—Fe1—C24	163.10 (7)	C24—C23—Fe1	69.52 (9)
C14—Fe1—C24	120.53 (7)	C22—C23—H23	125.5 (12)
C21—Fe1—C13	125.48 (7)	C24—C23—H23	126.2 (12)
C15—Fe1—C13	68.49 (7)	Fe1—C23—H23	124.0 (12)
C11—Fe1—C13	68.63 (7)	C25—C24—C23	108.49 (15)
C22—Fe1—C13	107.28 (7)	C25—C24—Fe1	69.55 (9)
C25—Fe1—C13	163.08 (7)	C23—C24—Fe1	69.92 (10)
C12—Fe1—C13	40.42 (7)	C25—C24—H24	126.2 (11)
C14—Fe1—C13	40.40 (8)	C23—C24—H24	125.1 (11)
C24—Fe1—C13	155.15 (7)	Fe1—C24—H24	122.7 (11)
C21—Fe1—C23	68.47 (6)	C24—C25—C21	107.56 (14)
C15—Fe1—C23	125.14 (7)	C24—C25—Fe1	70.04 (9)
C11—Fe1—C23	162.78 (7)	C21—C25—Fe1	68.79 (9)
C22—Fe1—C23	40.52 (7)	C24—C25—H25	125.2 (11)
C25—Fe1—C23	68.35 (7)	C21—C25—H25	127.0 (11)
C12—Fe1—C23	154.95 (7)	Fe1—C25—H25	122.4 (11)
C14—Fe1—C23	107.61 (7)	O22—C26—O21	123.69 (15)
C24—Fe1—C23	40.57 (7)	O22—C26—C21	123.47 (15)
C13—Fe1—C23	120.33 (7)	O21—C26—C21	112.83 (14)
C26—O21—H21	107.7 (17)	C2—N1—H10	117.7 (18)
C12—C11—C15	107.33 (15)	C2—N1—H11	119.6 (15)
C12—C11—C16	126.40 (15)	H10—N1—H11	117 (2)
C15—C11—C16	126.26 (15)	C1—N2—C5	122.83 (17)
C12—C11—Fe1	69.91 (9)	C1—N2—H2	118.5 (14)
C15—C11—Fe1	69.29 (9)	C5—N2—H2	118.7 (14)
C16—C11—Fe1	125.12 (11)	N2—C1—C2	120.94 (16)
C13—C12—C11	108.29 (16)	N2—C1—H1	115.5 (11)
C13—C12—Fe1	70.01 (10)	C2—C1—H1	123.6 (11)
C11—C12—Fe1	69.15 (9)	N1—C2—C1	120.30 (17)
C13—C12—H12	127.9 (12)	N1—C2—C3	122.88 (18)
C11—C12—H12	123.8 (12)	C1—C2—C3	116.76 (17)
Fe1—C12—H12	125.8 (12)	C4—C3—C2	120.22 (18)
C12—C13—C14	108.35 (16)	C4—C3—H3	120.3 (13)
C12—C13—Fe1	69.57 (10)	C2—C3—H3	119.4 (13)
C14—C13—Fe1	69.72 (10)	C5—C4—C3	120.33 (17)
C12—C13—H13	124.4 (13)	C5—C4—H4	119.4 (14)
C14—C13—H13	127.2 (13)	C3—C4—H4	120.3 (14)
Fe1—C13—H13	124.1 (13)	N2—C5—C4	118.91 (18)
C13—C14—C15	108.20 (16)	N2—C5—H5	116.4 (13)
C13—C14—Fe1	69.88 (10)	C4—C5—H5	124.7 (13)
C15—C14—Fe1	69.04 (10)		
			
C21—Fe1—C11—C12	82.37 (11)	C14—Fe1—C21—C25	−161.7 (2)
C15—Fe1—C11—C12	−118.52 (14)	C24—Fe1—C21—C25	−37.55 (10)
C22—Fe1—C11—C12	47.8 (2)	C13—Fe1—C21—C25	166.01 (10)
C25—Fe1—C11—C12	125.35 (10)	C23—Fe1—C21—C25	−81.32 (10)
C14—Fe1—C11—C12	−80.70 (11)	C15—Fe1—C21—C22	166.56 (15)
C24—Fe1—C11—C12	166.16 (10)	C11—Fe1—C21—C22	−158.87 (10)
C13—Fe1—C11—C12	−37.20 (11)	C25—Fe1—C21—C22	119.10 (14)
C23—Fe1—C11—C12	−161.9 (2)	C12—Fe1—C21—C22	−115.91 (10)
C21—Fe1—C11—C15	−159.11 (10)	C14—Fe1—C21—C22	−42.6 (3)
C22—Fe1—C11—C15	166.30 (15)	C24—Fe1—C21—C22	81.55 (10)
C25—Fe1—C11—C15	−116.13 (10)	C13—Fe1—C21—C22	−74.89 (12)
C12—Fe1—C11—C15	118.52 (14)	C23—Fe1—C21—C22	37.79 (10)
C14—Fe1—C11—C15	37.82 (10)	C15—Fe1—C21—C26	−74.4 (2)
C24—Fe1—C11—C15	−75.32 (12)	C11—Fe1—C21—C26	−39.84 (15)
C13—Fe1—C11—C15	81.31 (11)	C22—Fe1—C21—C26	119.03 (17)
C23—Fe1—C11—C15	−43.4 (3)	C25—Fe1—C21—C26	−121.87 (17)
C21—Fe1—C11—C16	−38.63 (16)	C12—Fe1—C21—C26	3.12 (14)
C15—Fe1—C11—C16	120.48 (18)	C14—Fe1—C21—C26	76.5 (3)
C22—Fe1—C11—C16	−73.2 (2)	C24—Fe1—C21—C26	−159.42 (15)
C25—Fe1—C11—C16	4.35 (15)	C13—Fe1—C21—C26	44.14 (16)
C12—Fe1—C11—C16	−121.01 (18)	C23—Fe1—C21—C26	156.82 (15)
C14—Fe1—C11—C16	158.29 (16)	C25—C21—C22—C23	−0.07 (18)
C24—Fe1—C11—C16	45.16 (17)	C26—C21—C22—C23	−174.85 (14)
C13—Fe1—C11—C16	−158.21 (16)	Fe1—C21—C22—C23	−60.00 (11)
C23—Fe1—C11—C16	77.1 (3)	C25—C21—C22—Fe1	59.92 (11)
C15—C11—C12—C13	−0.16 (19)	C26—C21—C22—Fe1	−114.85 (15)
C16—C11—C12—C13	178.69 (15)	C21—Fe1—C22—C23	118.68 (14)
Fe1—C11—C12—C13	59.27 (12)	C15—Fe1—C22—C23	−43.2 (3)
C15—C11—C12—Fe1	−59.42 (11)	C11—Fe1—C22—C23	166.96 (14)
C16—C11—C12—Fe1	119.42 (16)	C25—Fe1—C22—C23	80.76 (10)
C21—Fe1—C12—C13	124.47 (11)	C12—Fe1—C22—C23	−158.90 (10)
C15—Fe1—C12—C13	−81.27 (12)	C14—Fe1—C22—C23	−75.76 (12)
C11—Fe1—C12—C13	−119.72 (15)	C24—Fe1—C22—C23	37.27 (10)
C22—Fe1—C12—C13	81.25 (13)	C13—Fe1—C22—C23	−116.74 (11)
C25—Fe1—C12—C13	166.16 (11)	C15—Fe1—C22—C21	−161.9 (2)
C14—Fe1—C12—C13	−37.32 (11)	C11—Fe1—C22—C21	48.28 (19)
C24—Fe1—C12—C13	−161.8 (2)	C25—Fe1—C22—C21	−37.92 (9)
C23—Fe1—C12—C13	47.7 (2)	C12—Fe1—C22—C21	82.42 (11)
C21—Fe1—C12—C11	−115.81 (10)	C14—Fe1—C22—C21	165.56 (10)
C15—Fe1—C12—C11	38.45 (10)	C24—Fe1—C22—C21	−81.42 (10)
C22—Fe1—C12—C11	−159.02 (10)	C13—Fe1—C22—C21	124.58 (10)
C25—Fe1—C12—C11	−74.12 (12)	C23—Fe1—C22—C21	−118.68 (14)
C14—Fe1—C12—C11	82.40 (11)	C21—C22—C23—C24	0.38 (18)
C24—Fe1—C12—C11	−42.0 (3)	Fe1—C22—C23—C24	−58.70 (12)
C13—Fe1—C12—C11	119.72 (15)	C21—C22—C23—Fe1	59.08 (11)
C23—Fe1—C12—C11	167.44 (14)	C21—Fe1—C23—C22	−38.38 (10)
C11—C12—C13—C14	0.4 (2)	C15—Fe1—C23—C22	164.92 (10)
Fe1—C12—C13—C14	59.12 (12)	C11—Fe1—C23—C22	−161.4 (2)
C11—C12—C13—Fe1	−58.73 (12)	C25—Fe1—C23—C22	−82.70 (10)
C21—Fe1—C13—C12	−74.75 (13)	C12—Fe1—C23—C22	47.3 (2)
C15—Fe1—C13—C12	82.19 (11)	C14—Fe1—C23—C22	123.37 (10)
C11—Fe1—C13—C12	37.67 (10)	C24—Fe1—C23—C22	−119.96 (14)
C22—Fe1—C13—C12	−116.48 (11)	C13—Fe1—C23—C22	81.10 (12)
C25—Fe1—C13—C12	−41.7 (3)	C21—Fe1—C23—C24	81.58 (10)
C14—Fe1—C13—C12	119.72 (15)	C15—Fe1—C23—C24	−75.12 (12)
C24—Fe1—C13—C12	167.49 (15)	C11—Fe1—C23—C24	−41.5 (3)
C23—Fe1—C13—C12	−158.72 (10)	C22—Fe1—C23—C24	119.96 (14)
C21—Fe1—C13—C14	165.53 (10)	C25—Fe1—C23—C24	37.26 (9)
C15—Fe1—C13—C14	−37.53 (10)	C12—Fe1—C23—C24	167.30 (15)
C11—Fe1—C13—C14	−82.05 (11)	C14—Fe1—C23—C24	−116.67 (11)
C22—Fe1—C13—C14	123.80 (11)	C13—Fe1—C23—C24	−158.94 (10)
C25—Fe1—C13—C14	−161.4 (2)	C22—C23—C24—C25	−0.55 (19)
C12—Fe1—C13—C14	−119.72 (15)	Fe1—C23—C24—C25	−59.03 (11)
C24—Fe1—C13—C14	47.8 (2)	C22—C23—C24—Fe1	58.48 (11)
C23—Fe1—C13—C14	81.56 (12)	C21—Fe1—C24—C25	38.14 (10)
C12—C13—C14—C15	−0.5 (2)	C15—Fe1—C24—C25	−116.18 (10)
Fe1—C13—C14—C15	58.56 (12)	C11—Fe1—C24—C25	−74.16 (12)
C12—C13—C14—Fe1	−59.03 (12)	C22—Fe1—C24—C25	82.56 (10)
C21—Fe1—C14—C13	−42.2 (3)	C12—Fe1—C24—C25	−41.5 (3)
C15—Fe1—C14—C13	119.78 (15)	C14—Fe1—C24—C25	−158.78 (10)
C11—Fe1—C14—C13	81.48 (11)	C13—Fe1—C24—C25	167.36 (15)
C22—Fe1—C14—C13	−75.05 (13)	C23—Fe1—C24—C25	119.78 (14)
C25—Fe1—C14—C13	167.24 (15)	C21—Fe1—C24—C23	−81.64 (10)
C12—Fe1—C14—C13	37.33 (11)	C15—Fe1—C24—C23	124.04 (10)
C24—Fe1—C14—C13	−158.82 (10)	C11—Fe1—C24—C23	166.06 (10)
C23—Fe1—C14—C13	−116.40 (11)	C22—Fe1—C24—C23	−37.23 (10)
C21—Fe1—C14—C15	−162.0 (2)	C25—Fe1—C24—C23	−119.78 (14)
C11—Fe1—C14—C15	−38.30 (10)	C12—Fe1—C24—C23	−161.3 (2)
C22—Fe1—C14—C15	165.18 (10)	C14—Fe1—C24—C23	81.43 (12)
C25—Fe1—C14—C15	47.5 (2)	C13—Fe1—C24—C23	47.6 (2)
C12—Fe1—C14—C15	−82.44 (11)	C23—C24—C25—C21	0.50 (18)
C24—Fe1—C14—C15	81.40 (12)	Fe1—C24—C25—C21	−58.76 (11)
C13—Fe1—C14—C15	−119.78 (15)	C23—C24—C25—Fe1	59.26 (11)
C23—Fe1—C14—C15	123.83 (11)	C22—C21—C25—C24	−0.27 (18)
C13—C14—C15—C11	0.36 (19)	C26—C21—C25—C24	174.35 (15)
Fe1—C14—C15—C11	59.45 (11)	Fe1—C21—C25—C24	59.54 (11)
C13—C14—C15—Fe1	−59.09 (12)	C22—C21—C25—Fe1	−59.81 (11)
C12—C11—C15—C14	−0.13 (18)	C26—C21—C25—Fe1	114.81 (16)
C16—C11—C15—C14	−178.98 (15)	C21—Fe1—C25—C24	−119.03 (14)
Fe1—C11—C15—C14	−59.94 (11)	C15—Fe1—C25—C24	81.66 (11)
C12—C11—C15—Fe1	59.82 (11)	C11—Fe1—C25—C24	124.90 (10)
C16—C11—C15—Fe1	−119.03 (16)	C22—Fe1—C25—C24	−81.02 (11)
C21—Fe1—C15—C14	166.96 (14)	C12—Fe1—C25—C24	166.22 (10)
C11—Fe1—C15—C14	118.87 (15)	C14—Fe1—C25—C24	47.9 (2)
C22—Fe1—C15—C14	−42.6 (3)	C13—Fe1—C25—C24	−161.6 (2)
C25—Fe1—C15—C14	−159.02 (10)	C23—Fe1—C25—C24	−37.39 (10)
C12—Fe1—C15—C14	80.74 (11)	C15—Fe1—C25—C21	−159.31 (9)
C24—Fe1—C15—C14	−116.75 (11)	C11—Fe1—C25—C21	−116.08 (10)
C13—Fe1—C15—C14	37.21 (11)	C22—Fe1—C25—C21	38.01 (10)
C23—Fe1—C15—C14	−75.53 (13)	C12—Fe1—C25—C21	−74.75 (11)
C21—Fe1—C15—C11	48.1 (2)	C14—Fe1—C25—C21	166.90 (15)
C22—Fe1—C15—C11	−161.4 (2)	C24—Fe1—C25—C21	119.03 (14)
C25—Fe1—C15—C11	82.11 (11)	C13—Fe1—C25—C21	−42.6 (3)
C12—Fe1—C15—C11	−38.13 (10)	C23—Fe1—C25—C21	81.64 (10)
C14—Fe1—C15—C11	−118.87 (15)	C25—C21—C26—O22	−170.69 (15)
C24—Fe1—C15—C11	124.38 (10)	C22—C21—C26—O22	3.1 (2)
C13—Fe1—C15—C11	−81.66 (11)	Fe1—C21—C26—O22	−83.00 (18)
C23—Fe1—C15—C11	165.60 (9)	C25—C21—C26—O21	10.2 (2)
C12—C11—C16—O11	14.1 (2)	C22—C21—C26—O21	−176.01 (15)
C15—C11—C16—O11	−167.29 (15)	Fe1—C21—C26—O21	97.89 (14)
Fe1—C11—C16—O11	103.88 (16)	C5—N2—C1—C2	−1.0 (3)
C12—C11—C16—O12	−167.90 (15)	N2—C1—C2—N1	−176.66 (18)
C15—C11—C16—O12	10.7 (2)	N2—C1—C2—C3	0.5 (3)
Fe1—C11—C16—O12	−78.10 (18)	N1—C2—C3—C4	177.35 (19)
C15—Fe1—C21—C25	47.46 (19)	C1—C2—C3—C4	0.3 (3)
C11—Fe1—C21—C25	82.02 (11)	C2—C3—C4—C5	−0.6 (3)
C22—Fe1—C21—C25	−119.10 (14)	C1—N2—C5—C4	0.7 (3)
C12—Fe1—C21—C25	124.98 (10)	C3—C4—C5—N2	0.1 (3)

### Hydrogen-bond geometry (Å, º)

**Table:**

*D*—H···*A*	*D*—H	H···*A*	*D*···*A*	*D*—H···*A*
N1—H11···O12	0.87 (3)	2.08 (3)	2.918 (2)	161 (2)
N1—H10···O11^i^	0.84 (3)	2.07 (3)	2.906 (2)	171 (3)
N2—H2···O11^ii^	0.89 (2)	1.79 (2)	2.675 (2)	177 (2)
O21—H21···O12^iii^	0.81 (2)	1.77 (2)	2.5621 (16)	164 (2)

Symmetry codes: (i) −*x*, −*y*+1, −*z*+1; (ii) −*x*, *y*+1/2, −*z*+1/2; (iii) *x*, −*y*+1/2, *z*+1/2.
